# Density Functional Theory of Polymer Structure and Conformations

**DOI:** 10.3390/polym8040121

**Published:** 2016-04-15

**Authors:** Zhaoyang Wei, Nanying Ning, Liqun Zhang, Ming Tian, Jianguo Mi

**Affiliations:** 1 State Key Laboratory of Organic-Inorganic Composites, Beijing University of Chemical Technology, Beijing 100029, China; 2014400083@mail.buct.edu.cn (Z.W.); ningny@mail.buct.edu.cn (N.N.); zhanglq@mail.buct.edu.cn (L.Z.); 2 Key Laboratory of Beijing City on Preparation and Processing of Novel Polymer Materials, Beijing University of Chemical Technology, Beijing 100029, China

**Keywords:** density functional theory, scaling exponent, radius of gyration

## Abstract

We present a density functional approach to quantitatively evaluate the microscopic conformations of polymer chains with consideration of the effects of chain stiffness, polymer concentration, and short chain molecules. For polystyrene (PS), poly(ethylene oxide) (PEO), and poly(methyl methacrylate) (PMMA) melts with low-polymerization degree, as chain length increases, they display different stretching ratios and show non-universal scaling exponents due to their different chain stiffnesses. In good solvent, increase of PS concentration induces the decline of gyration radius. For PS blends containing short ($m_{1}=1-100$) and long (m=100) chains, the expansion of long chains becomes unobvious once $m_{1}$ is larger than 40, which is also different to the scaling properties of ideal chain blends.

## 1. Introduction

The description of macromolecular conformations in various environments is an outstanding problem in polymer physics [1,2,3,4,5,6,7,8]. The scaling properties may be altered in different media and polymer ratios. Theoretical concepts of polymer physics have frequently been used to establish the connection between polymer microstructure and dimension [9,10,11]. On the other hand, the advances in experimental techniques, such as neutron scattering methods, allow a direct measurement of the intramolecular and intermolecular structure, thus providing, in principle, the possibility of testing these theories [11,12,13,14].

The stretching of polymers has been treated exhaustively by a number of Flory-type mean field theories based on general principles that relate the dimensions and the length of a chain to its free-energy [15,16,17,18]. These theories provide reasonable scaling exponent that correlate the gyration radius ($R_{g}$) of polymer chain to its length (N) via $R_{g}\propto N^{v}$. A prerequisite for application of these theories is that $R_{g}$ at θ-state are known, which serves as an essential reference state. Although the scaling exponent at θ-state has been determined as ν=1/2, the pre-coefficient of the relational expression is still unavailable. Moreover, since the free-energy expression is independent of microscopic inter- and intramolecular structures, the quantitative application of these theories and their comparison to experimental results in different conditions are still far from satisfactory.

Polymer reference interaction site model (PRISM) integral equation provides a framework for describing the microscopic structures of uniform polymeric fluids [19,20]. The intermolecular correlation functions are calculated for a given set of intramolecular correlation functions after the equation is formulated and coupled with a closure relation. It has been successfully applied to describe the intermolecular structure of a broad range of polymeric systems [21,22,23]. Since the intramolecular correlation function is typically unknown, the equation suffers from a serious self-consistency problem. Although this problem can be partially solved by involving a semiflexible chain model or inserting a single-chain molecular simulation to integrate the intra- and intermolecular correlation functions, this strategy is restricted to improve the accuracy of intermolecular correlation function, whereas the intramolecular correlation function cannot be derived from the equation. Besides, the integral equation approach suffers inconsistency among different routes for thermodynamic properties. As such, the structure description provided by the PRISM is generally qualitative. In order to present a quantitative evaluation to the intramolecular correlation function, an explicit free-energy analysis of the system is undoubtedly necessary.

Classical density functional theory (DFT) provides another route to represent both structural and thermodynamic properties of polymeric fluids [24,25,26]. The theory for fluids is based on the minimization of grand free-energy functional, and gives prediction for the equilibrium free-energy and microscopic structure of the components. In particular, the theory yields self-contained structural and thermodynamic properties and employs no molecular simulations as input. In recent years, an accurate density functional approach has been developed for polymer systems by combining the modified interfacial statistical association fluid theory [27,28,29,30]. The excess Helmholtz free-energy due to polymerization is related to the association equilibrium through the multibody cavity correlation functions. In order to characterize the intramolecular correlation function, a test-particle method has been integrated into a relatively simple DFT to calculate the local inhomogeneous density profile of the polymeric fluid in the external field of one segment fixed at the origin [31,32]. Such DFT is based on the first order thermodynamic perturbation theory for polyatomic molecules. It avoids molecular simulations as input and shows the advantages of self-consistency among inter- and intramolecular correlation functions, and is very accurate in comparison with simulation data for freely jointed hard-sphere chains. The drawback of the theory lies in its ideal chain model. In fact, different polymers in experiments have different degrees of stiffness to their backbones.

There is considerable interest in developing a theory capable of accurately predicting the microscopic conformation of complicated polymer fluids to decipher the stretching properties. In this paper, we present a density functional approach by combing the PRISM equation, the test-particle method, and the modified interfacial statistical association fluid theory [32] to deal with actual polymer conformations, particularly the intramolecular correlation functions. The radii of gyration of polystyrene (PS), poly(ethylene oxide) (PEO), and poly(methyl methacrylate) (PMMA) are calculated to analyze their expansion or contraction under different conditions. We utilize coarse-graining chain for modeling the conformational behavior of the polymers with chain stiffness, reflecting the dependence of bending energy on the angle between two nearby bond or tangent vectors. Since all parameters are taken from the general force field [33,34,35], all results given by the theory are strict predictions.

The rest of the paper is organized as follows: Section 2 presents the whole molecular model and the DFT calculation method. Section 3 shows the calculation results of gyration radius and structure factor. The expansion effects are evaluated for polymer melts, concentration, and blends containing long and short chains. Section 4 summarizes the final conclusion.

## 2. Model and Approach

**Figure 1:**
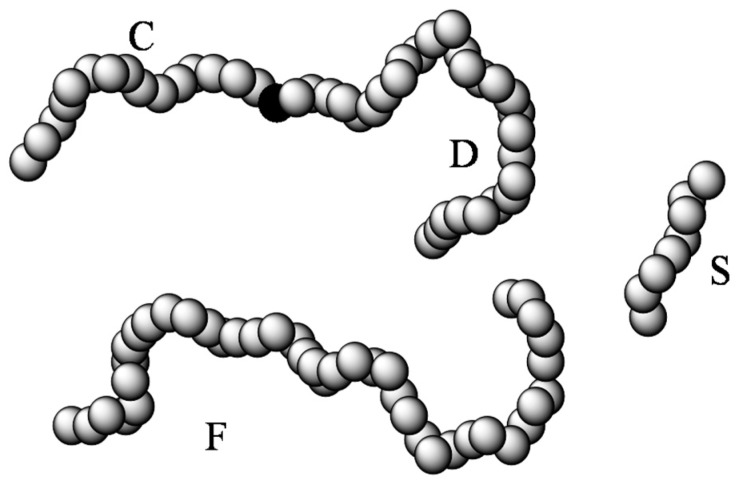
Schematic of the polymer model used in this work. Here, a middle segment from a polymer chain (filled black sphere) is fixed at the origin. The density distributions of segments from the tethered fragments (C and D), free (F), and short (S) chain molecules are related to the intra- and intermolecular segment–segment correlation functions. In a homogeneous polymer melt, the short chain no longer exists.

To simplify the complicated polymer conformation, we use a coarse-grained model to describe polymer chains. Each polymer chain is represented by a series of bonded segments and obeys the semiflexible chain conformation. The segment diameter is given by repeating unit. Suppose that one segment from an arbitrarily selected chain is fixed at the origin. The system considered is equivalent to a mixture of four polymeric components (F, S, C and D) in a symmetric external field due to the fixed segment, as depicted in Figure 1.

The free molecules are represented by F, composed of $m_{F}$ segments; the chemically identical short chain molecules are represent by S, composed of $m_{S}$ segments; while the tethered fragments are represented by C and D, composed of $m_{C}$ and $m_{D}$ segments, respectively. At equilibrium, the density distributions of free chains, short chains, and the tethered fragments satisfy the variational relations

(1) $$\frac{\delta \Omega }{{\delta \rho }_{i}^{\left(F\right)}(r)}=\frac{\delta \Omega }{{\delta \rho }_{i}^{\left(S\right)}(r)}=\frac{\delta \Omega }{{\delta \rho }_{i}^{\left(C\right)}(r)}=\frac{\delta \Omega }{{\delta \rho }_{i}^{\left(D\right)}(r)}=0$$

where Ω stands for the grand free-energy, $\rho_{i}^{\left(l\right)}(r)$ (l=F, S, C, and D) is the density of the ith segment on chain l at position r. For a homogeneous polymer melt, the contribution of the S component is automatically deleted.

The segment distributions of the free (F) and short (S) chains around the fixed segment are related to the intermolecular site–site correlation function $g_{ij}(r)=\rho_{i,j}(r)/\rho_{b}$, where $\rho_{i,j}(r)$ is the density profile of segments i on molecule F or S around the fixed segment j, and $\rho_{b}$ is the bulk density of segment. Whereas the distribution of segments from the fragments C and D are related to the intramolecular correlation function $\omega_{ij}(r)=\rho_{i,j}(r)$, where $\rho_{i,j}\left(r\right)$ is the density profile of segment i on the tethered chain (C or D) around the fixed segment j. There is only one tethered polymer chain, therefore the normalization condition is $\int \omega_{i,j}\left(r\right)dr=1$. As a consequence, the site–site intra- and intermolecular correlation functions specify all detail microscopic structures of a polymeric fluid. In particular, the gyration radius is a version of the intramolecular correlation function. From the site–site correlation functions, we can calculate the average intermolecular correlation function with $g\left(r\right)=\frac{1}{m_{F}^{2}}\sum_{i=1}^{m_{F}}\sum_{j=1}^{m_{F}}g_{ij}\left(r\right)$, and the average intramolecular correlation function with $\omega \left(r\right)=\frac{1}{m_{F}}\sum_{i=1}^{m_{F}}\sum_{j=1}^{m_{F}}\omega_{ij}\left(r\right)$.

The system considered above is equivalent to a mixture of four polymeric components (F+S+C+D) in a symmetric external field due to the fixed segment. The grand potential is related to the Helmholtz free-energy A[ρ(r)] through the Legendre transform

(2) $$\Omega \left[\rho_{i}^{\left(F\right)}\left(r\right),\rho_{i}^{\left(S\right)}\left(r\right),\rho_{i}^{\left(C\right)}\left(r\right),\rho_{i}^{\left(D\right)}\left(r\right)\right]=A\left[\rho_{i}^{\left(F\right)}\left(r\right),\rho_{i}^{\left(S\right)}\left(r\right),\rho_{i}^{\left(C\right)}\left(r\right),\rho_{i}^{\left(D\right)}\left(r\right)\right]-\sum_{l=F,S,C,D}\sum_{i=1}^{m_{l}}\int dr^{\prime }(\mu_{i}^{\left(l\right)}-V_{\text{ext}}^{i,l}(r^{\prime }))\rho_{i}^{\left(l\right)}\left(r^{\prime }\right)$$

where $\mu_{i}^{\left(l\right)}$ is the chemical potential of that segment i, $V_{\text{ext}}^{i,l}$ is the external field acting on that segment i. The first summation is over all chains l in the mixture (F,S,C,D), and the second summation is over all segments of chain l. For any segment that is not immediately bonded with the fixed segment, the external potential is identical to the Lennard–Jones (LJ) interaction potential. While for two segments that are directly connected to the fixed segment, the external potential includes the bonding potential $v_{\text{bond}}$, given by $\exp[-\beta v_{\text{bond}}(r_{i},r_{j})]=\delta (\left|r_{i}-r_{j}\right|-\sigma )/4{\pi \sigma }^{2}$; here segments i and j are nearest neighbors from the same molecule, and δ is the Dirac-delta function. The bond length (L), bond angle (θ), and nonbonded parameters (σ and ε) for PS, PEO, and PMMA are summarized in Table 1.

The total Helmholtz free-energy functional can be decomposed into ideal and excess contributions [27]. The ideal contribution is generally represented by $A^{\text{id}}=\int dr\sum \rho_{i}^{\left(l\right)}\left(r\right)\left[\ln\left[\rho_{i}^{\left(l\right)}\left(r\right)\right]-1\right]$. The excess contribution consists of hard-sphere repulsion, long-range attraction, chain stiffness, and chain connectivity over the ideal gas state of the atomic mixture.

The free-energy due to hard-sphere repulsion is given by the fundamental measure theory [24]

(3) $$A^{\text{hs}}\left[\rho_{i}^{\left(l\right)}(r)\right]=\int dr\Phi^{\text{hs}}[n_{\gamma }(r)]$$

where $\Phi^{\text{hs}}[n_{\gamma }(r)]$ is the free-energy density, which stems from the modified fundamental theory [36], including both the scalar and vector contributions

(4) $$\Phi^{\text{hs}}\left[n_{\gamma }\left(r\right)\right]=\left[-n_{0}\ln\left(1-n_{3}\right)+\frac{n_{1}n_{2}-n_{V_{1}}\cdot n_{V_{2}}}{1-n_{3}}+\frac{1}{36\pi }\left(n_{3}\ln\left(1-n_{3}\right)+\frac{n_{3}^{2}}{{\left(1-n_{3}\right)}^{2}}\right)\frac{n_{2}^{3}-3n_{2}n_{V_{2}}\cdot n_{V_{2}}}{n_{3}^{3}}\right]$$

where $n_{\gamma }(r)$ with $\gamma =0,\;1,\;2,\;3,\;V_{1},\;V_{2}$ are the weighted densities. The details have been given elsewhere [36].

The long-range attractive contribution to the free-energy functional can be simplified as

(5) $$A^{\text{att}}\left[\rho_{i}^{\left(l\right)}(r)\right]=\frac{1}{2}\sum_{i}\sum_{j}\int dr^{\prime }\int dr^{\prime \prime }\rho_{i}^{\left(l\right)}(r^{\prime })\rho_{j}^{\left(l\right)}(r^{\prime \prime })u_{ij}^{\text{att}}(\left|r^{\prime }-r^{\prime \prime }\right|)$$

where $u_{ij}^{\text{att}}(r)$ is the interaction potential between any two species i and j, and can be represented by a cut-and-shifted LJ potential with a Weeks–Chandler–Anderson separation [37,38].

The functional $A^{\text{stiff}}$ is constructed to account for the contribution of conformational entropy given by the polymer stiffness, which is implemented to improve the accuracy for description of real polymer chains

(6) $$A^{\text{stiff}}\left[\rho_{i}^{\left(l\right)}(r)\right]=-\frac{1}{2}\sum_{i}\sum_{j}\int dr^{\prime }\int dr^{\prime \prime }\rho_{i}^{\left(l\right)}(r^{\prime })\rho_{j}^{\left(l\right)}(r^{\prime \prime })c_{ij}^{\text{stiff}}(\left|r^{\prime }-r^{\prime \prime }\right|)\;$$

with the approximation $c^{\text{stiff}}(r)=c^{\text{semiflexible}}(r)-c^{\text{flexible}}(r)$. Here $c^{\text{semiflexible}}(r)$ and $c^{\text{flexible}}(r)$ denote the direct correlation functions of semiflexible and flexible polymer chains. They are calculated from the PRISM integral equation [39]

(7) $$h(r)=\int d{\vec{r}}^{\prime }\int d{\vec{r}}^{\prime \prime }\omega (|\vec{r}-{\vec{r}}^{\prime }|)c(|{\vec{r}}^{\prime }-{\vec{r}}^{\prime \prime }|)[\omega (r^{\prime \prime })+\rho h(r^{\prime \prime })]$$

where h(r) is the total correlation function and ω(r) is the intramolecular correlation function. ω(r) is cursorily represented by the Koyama model [40] for flexible or semiflexible chains. To solve the equation, we adopt the Kovalenko–Hirata approximation [41].

To compute the free-energy contribution due to chain connectivity, we use the Tripathi–Chapman functional [27], in which the chains are treated as a sequence of m bonded monomers, enforced by giving each segment a label and allowing segments to exclusively bond to their specific matching segments. It is indicated as

(8) $$A^{\text{chain}}\left[\rho_{i}^{\left(l\right)}(r)\right]=\int dr^{\prime }\sum_{i=1}^{m_{l}}\rho_{i}^{\left(l\right)}(r^{\prime })\times \sum_{A\in \Gamma^{\left(i\right)}}\left(\ln X_{A}^{i,l}(r^{\prime })-\frac{X_{A}^{i,l}(r^{\prime })}{2}+\frac{1}{2}\right)$$

in which the first summation is over all the segments i, and the second is over all the association sites on segment i as $\Gamma^{\left(i\right)}$, representing the set of all associating sites on segment i. $X_{A}^{i,l}$ is the fraction of segment i that are not bonded at their association site A.

(9) $$X_{A}^{i.l}(r)=\frac{1}{1+\int dr^{\prime }X_{B}^{j,l}(r^{\prime })\Delta_{ij}^{\left(l\right)}(r,r^{\prime })\rho_{j}^{\left(l\right)}(r^{\prime })}$$

where j denotes the neighboring segment that will bond to segment i, and $\Delta_{ij}^{\left(l\right)}\left(r,r^{\prime }\right)=KF_{ij}^{\left(l\right)}\left(r,r^{\prime }\right)y_{ij}^{\left(l\right)}\left(r,r^{\prime }\right)$. Here K is a geometric constant that accounts for the volume available for bonding between segments, and $F_{ij}^{\left(l\right)}\left(r,r^{\prime }\right)=\exp\left({\beta \varepsilon }_{0}-\beta v_{\text{bond}}^{ij}\left(r,r^{\prime }\right)\right)-1$ represents the association Mayer *f*-function. $y_{ij}^{\left(l\right)}\left(r,r^{\prime }\right)$ is the cavity correlation function. The details have been given elsewhere [27].

The functional derivatives of the free-energies are required to obtain the equilibrium density profiles, which are given as $\partial \Omega /\partial \rho_{i}^{\left(l\right)}\left(r\right)=0$. As a result, the Euler–Lagrange Equation is written as

(10) $$\ln\rho_{i}^{\left(l\right)}(r)+\sum_{A\in \Gamma^{\left(i\right)}}\ln X_{A}^{i,l}(r)-\frac{1}{2}\sum_{q=F,S,C,D}\sum_{\gamma =1}^{m_{q}}\sum_{\gamma \prime }^{\left\{\gamma \prime \right\}}\int \rho_{\gamma }^{\left(q\right)}(r^{\prime })\frac{\delta \ln y^{\gamma \gamma \prime }(r^{\prime })}{{\delta \rho }_{i}^{\left(l\right)}(r)}dr^{\prime }+\frac{\delta \beta A^{\text{hs}}}{{\delta \rho }_{i}^{\left(l\right)}(r)}+\frac{\delta \beta A^{\text{att}}}{{\delta \rho }_{i}^{\left(l\right)}(r)}+\frac{\delta \beta A^{\text{stiff}}}{{\delta \rho }_{i}^{\left(l\right)}(r)}=\beta (\mu_{i}^{\left(l\right)}-V_{\text{ext}}^{i,l}(r))$$

where $\left\{\gamma^{\prime }\right\}$ is the set of all segments bonded to segment γ. This equation can be rewritten to give the density profile

(11) $$\rho_{i}^{\left(l\right)}(r)=\exp\left({\beta \mu }_{M_{l}}\right)\exp\left[D_{i}^{\left(l\right)}(r)-\beta V_{\text{ext}}^{i,l}(r)\right]I_{1,i}^{\left(l\right)}(r)I_{2,i}^{\left(l\right)}(r)$$

with

(12) $$D_{i}^{\left(l\right)}(r)=\frac{1}{2}\sum_{q=F,S,C,D}\sum_{\gamma =1}^{m_{q}}\sum_{\gamma \prime }^{\left\{\gamma \prime \right\}}\int \rho_{\gamma }^{\left(q\right)}(r^{\prime })\frac{\delta \ln y^{\gamma \gamma \prime }(r^{\prime })}{{\delta \rho }_{i}^{\left(l\right)}(r)}dr^{\prime }-\frac{\delta \beta A^{\text{hs}}}{{\delta \rho }_{i}^{\left(l\right)}(r)}-\frac{\delta \beta A^{\text{att}}}{{\delta \rho }_{i}^{\left(l\right)}(r)}-\frac{\delta \beta A^{\text{stiff}}}{{\delta \rho }_{i}^{\left(l\right)}(r)}$$

In Equation (11), $\mu_{M_{l}}$ is the bulk chemical potential of chain l. The multiple integrals $I_{1,i}^{\left(l\right)}(r)\;$ and $\;I_{2,i}^{\left(l\right)}(r)$ for the free and short chains (l=F,S) are solved in a recursive fashion and are given by

(13) $$\left\{ \begin{array}{l} I_{1,1}^{\left(l\right)}(r)=1\\ I_{1,i}^{\left(l\right)}(r)=\int I_{1,i-1}^{\left(l\right)}(r^{\prime })\exp\left[D_{i-1}^{\left(l\right)}(r^{\prime })-\beta V_{\text{ext}}^{i-1,l}(r^{\prime })\right]\Delta_{i-1,i}^{\left(l\right)}(r^{\prime },r)\left(\frac{r^{\prime }\Theta \left(\sigma -\left|r^{\prime }-r\right|\right)}{r}\right)dr^{\prime } \end{array}\right.$$

(14) $$\left\{ \begin{array}{l} I_{2,i}^{\left(l\right)}(r)=\int I_{2,i+1}^{\left(l\right)}(r^{\prime })\exp\left[D_{i+1}^{\left(l\right)}(r^{\prime })-\beta V_{\text{ext}}^{i+1,l}(r^{\prime })\right]\Delta_{i,i+1}^{\left(l\right)}(r,r^{\prime })\left(\frac{r^{\prime }\Theta \left(\sigma -\left|r^{\prime }-r\right|\right)}{r}\right)dr^{\prime }\\ I_{2,m_{F}}^{\left(l\right)}(r)=1 \end{array}\right.$$

where Θ(r) is the Heaviside step function. When applied to the tethered chains (l=C,D), $I_{1,i}^{\left(l\right)}(r)\;$ and $I_{2,i}^{\left(l\right)}(r)$ can be expressed as

(15) $$\left\{ \begin{array}{l} I_{1,1}^{\left(l\right)}(\sigma )=1\\ I_{1,2}^{\left(l\right)}(r)=\exp\left[D_{1}^{\left(l\right)}(\sigma )-\beta V_{\text{ext}}^{1,l}(\sigma )\right]\Delta_{1,2}^{\left(l\right)}(\sigma ,r)\left(\frac{\sigma \Theta \left(\sigma -\left|\sigma -r\right|\right)}{r}\right)\\ I_{1,i}^{\left(l\right)}(r)=\int I_{1,i-1}^{\left(l\right)}(r^{\prime })\exp\left[D_{i-1}^{\left(l\right)}(r^{\prime })-\beta V_{\text{ext}}^{i-1,l}(r^{\prime })\right]\Delta_{i-1,i}^{\left(l\right)}(r^{\prime },r)\left(\frac{r^{\prime }\Theta \left(\sigma -\left|r^{\prime }-r\right|\right)}{r}\right)dr^{\prime } \end{array}\right.$$

(16) $$\left\{ \begin{array}{l} I_{2,1}^{\left(l\right)}(\sigma )=\int I_{2,2}^{\left(l\right)}(r^{\prime })\exp\left[D_{2}^{\left(l\right)}(r^{\prime })-\beta V_{\text{ext}}^{2,l}(r^{\prime })\right]\Delta_{1,2}^{\left(l\right)}(\sigma ,r^{\prime })\left(\frac{r^{\prime }\Theta \left(\sigma -\left|r^{\prime }-\sigma \right|\right)}{\sigma }\right)dr^{\prime }\\ I_{2,i}^{\left(l\right)}(r)=\int I_{2,i+1}^{\left(l\right)}(r^{\prime })\exp\left[D_{i+1}^{\left(l\right)}(r^{\prime })-\beta V_{\text{ext}}^{i+1,l}(r^{\prime })\right]\Delta_{i,i+1}^{\left(l\right)}(r,r^{\prime })\left(\frac{r^{\prime }\Theta \left(\sigma -\left|r^{\prime }-r\right|\right)}{r}\right)dr^{\prime }\\ I_{2,m_{l}}^{\left(l\right)}(r)=1 \end{array}\right.$$

The chemical potentials of the tethered fragments C and D can be determined using the normalization conditions $\int 4\pi r^{2}\rho_{i}^{\left(C\right)}(r)dr=1$ and $\int 4\pi r^{2}\rho_{i}^{\left(D\right)}(r)dr=1$, where $i=1,2,3,...m_{l},\;\mathrm{for}\;l=C\;\mathrm{or}\;D$. For the first tethered segment (segment “1” of l=C or D),

(17) $$\int 4\pi r^{2}\left\{\exp\left(\beta \mu_{M_{l}}\right)\exp\left[D_{1}^{\left(l\right)}(r)-\beta V_{\text{ext}}^{1,l}(r)\right]I_{1,1}^{\left(l\right)}(r)I_{2,1}^{\left(l\right)}(r)\right\}dr=1$$

which yields $\exp({\beta \mu }_{M_{l}})=1/[\exp(D_{1}^{\left(l\right)}(\sigma ))I_{1,1}^{\left(l\right)}(\sigma )I_{2,1}^{\left(l\right)}(\sigma )]$. Using other segments yields equivalent results for $\mu_{M_{l}}$. Substituting this into Equation (11), we obtain

(18) $$\rho_{1}^{\left(C\right)}\left(r\right)=\rho_{1}^{\left(D\right)}\left(r\right)=\frac{\delta \left(r-\sigma \right)}{4{\pi \sigma }^{2}}$$

which matches the known condition for the tethered segment. Solving for the other segments $\left(i=2,3,...m_{l}\right)$ gives

(19) $$\rho_{i}^{\left(l\right)}(r)=\frac{1}{\exp\left[D_{1}^{\left(l\right)}(\sigma )\right]I_{1,1}^{\left(l\right)}(\sigma )I_{2,1}^{\left(l\right)}(\sigma )}\exp\left[D_{i}^{\left(l\right)}(r)-\beta V_{\text{ext}}^{i,l}(r)\right]I_{1,i}^{\left(l\right)}(r)I_{2,i}^{\left(l\right)}(r)$$

In calculating inter- and intramolecular correlation functions, we fix the segments of a polymer chain one by one and the density distributions around the fixed segment are calculated with Equations (11)–(19). Because of symmetry, $m_{F}/2$ (if $m_{F}$ is even) or $\left(m_{F}+1\right)/2$ (if $m_{F}$ is odd) calculations are required for predicting the detailed local structures of polymer chains consisting of $m_{F}$ identical segments. The density profiles are solved using the Picard-type iterative method. In the theoretical calculations, the computation domain is divided into equally-spaced grid points along the dimension normal to the surface. The grid spacing of 0.02σ is used in our calculation. At every iteration step, a new estimate to the density profiles is calculated with Equation (11), and then is mixed with the old one. The result acts as the new guess for the next iteration. This procedure is iterated until numerical self-consistency is achieved, in the sense that the difference of density profile between the preceding step and the present step is less than $5.0\times 10^{-4}$. All the convolution results of the equations are directly evaluated in the real space. In order to save computational time, we focus on those low-polymerized systems, where the monomer number is not more than 120. Unless noted otherwise, all calculations are performed at a total packing fraction of 0.40, which corresponds to a typical dense melt value with a realistic dimensionless isothermal compressibility.

## 3. Results and Discussion

**Figure 2:**
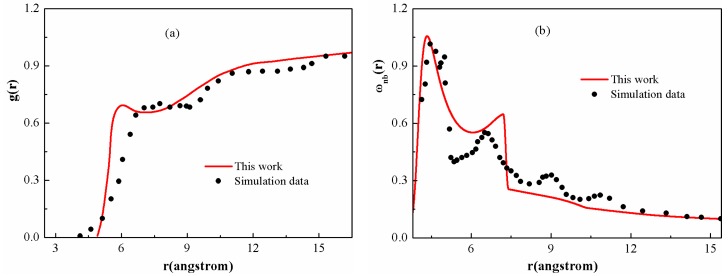
Comparison of the average (**a**) inter- and (**b**) intramolecular correlation functions of the PS chain obtained from theory and molecular simulations [42] at 413.2 K.

Figure 2 presents the inter- and intramolecular correlation functions for the PS chain given by the DFT, along with the simulation results that are attainable from the literature [42]. It is shown that the overall shape of theoretical curves and the positions of the main peaks can match the simulation results, indicating that the current theoretical model is suitable to quantitatively evaluate the microscopic structure of polymer melts. The deviations are probably due to the approximation of the model, in which the tacticity or torsional angle effects are overlooked. As shown in Figure 2a, on short length scales, the local structure of the PS chain is seen to have “liquid-like” tendency, displaying shells of first-nearest neighbors. Over longer distances, the curves exhibit a spatially slowly varying “correlation hole”, corresponding to the relative absence of neighboring sites due to intramolecular screening. Figure 2b presents the corresponding “nonbonded” intramolecular correlation functions. The discontinuity at r=7 Å is due to the direct interaction between next nearest neighbors along the polymer chain.

**Figure 3:**
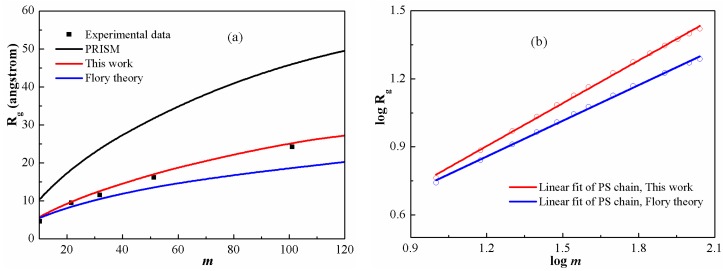
(**a**) Gyration radius as a function of chain length for PS chains at 500 K; (**b**) Determination of the scaling exponents. The circles are calculated results and the solid line is the best linear regression of the circles.

As the essential criterion to evaluate polymer structure and dimension, the gyration radius $R_{g}$ can be calculated from the intramolecular correlation function [40,43]

(20) $$R_{g}=\sqrt{\frac{2\pi }{m}\int_{0}^{\infty }r^{4}\omega \left(r\right)dr}$$

to quantitatively analyze polymer conformation. Figure 3a shows $R_{g}$ for PS chains at T=500 K as function of chain length in comparison with the available experimental data [44]. It is clear that both the PRISM equation and the Flory theory cannot provide reasonable intramolecular structure description. In contrast, a good agreement has been achieved between DFT calculations and experimental values in the whole range of chain length. In particular, the scaling exponents derived from the Flory theory and the DFT are fitted in Figure 3b. The values are 0.50 and 0.63, respectively. It shows clearly that, at a low degree of polymerization, the exponent from DFT is obviously higher than the universal value (0.50). In other words, actual polymers at a low degree of polymerization display non-universal scaling behavior and have a relatively larger scaling exponent, which can be attributed to their rigid characteristics. Moreover, as the chain becomes stiffer, the excluded volume increases, leading to increasing $R_{g}$. A similar result was also derived from molecular simulations [45,46].

Another consequence of the gradual ramp-up of polymer stiffness manifests itself through the form factor, which is particularly useful for studying the scaling behavior. Generally, the form factor is derived from light scattering experiments. In the theoretical model, it can be directly calculated from the Fourier transform of the intramolecular correlation function [47]

(21) $$P(q)=\frac{1}{m^{2}}\sum_{i=1}^{m}\sum_{j=1}^{m}\frac{\sin(qr_{ij})}{qr_{ij}}=\frac{1}{m^{2}}\sum_{i=1}^{m}\sum_{j=1}^{m}\omega_{ij}(q)=\frac{1}{m}\omega (q)$$

where $r_{ij}$ is the distance between segments i and j, and q denotes the magnitude of the scattering wave vector q. The expression for the form factor of a free chain is the well-known Debye equation

(22) $$P(q)=\frac{2}{{\left(q^{2}R_{g}^{2}\right)}^{2}}(\exp(-q^{2}R_{g}^{2})-1+q^{2}R_{g}^{2})$$

**Figure 4:**
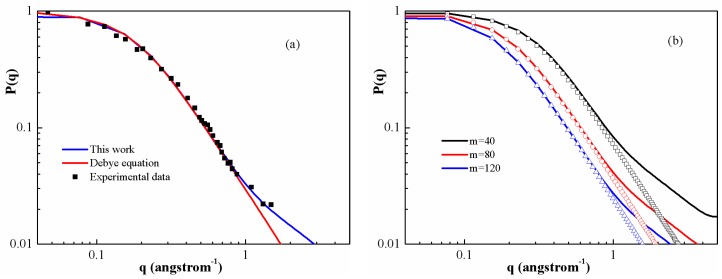
(**a**) Comparison between experimental data of the form factors and calculated ones for PS melt with the molecular weight 1 × 10^4^ in θ solvent; (**b**) Form factor for different lengths of PS melt at 500 K. Solid lines correspond to DFT calculations in Equation (21). Symbols show the curve obtained from the Debye expression.

Figure 4a shows the form factors for PS melt with molecular weight 1 × 10^4^ in θ solvent. It seems that a good agreement can be achieved between DFT calculations and the experimental values [48], whereas the Debye expression is not always valid in the fractal regime (q≥1.0). This can be seen by the gradual transition from a slope of −2 for the Debye equation to a slope of −1 for PS chains. Figure 4b shows the form factor of several different lengths of PS melt. In particular, we are interested in the so-called fractal regime. This regime provides information related to the chain statistics inside the coil, which can reveal details about stiffness and self-avoiding behavior. In the regime, the results given by two equations are closer as the polymerization degree increases. One can expect that on a scale which is large enough, they will again appear as flexible coils.

A standard way to examine the structure of a polymeric chain at all distances is through the static structure factor S(q), which is defined as

**Figure 5:**
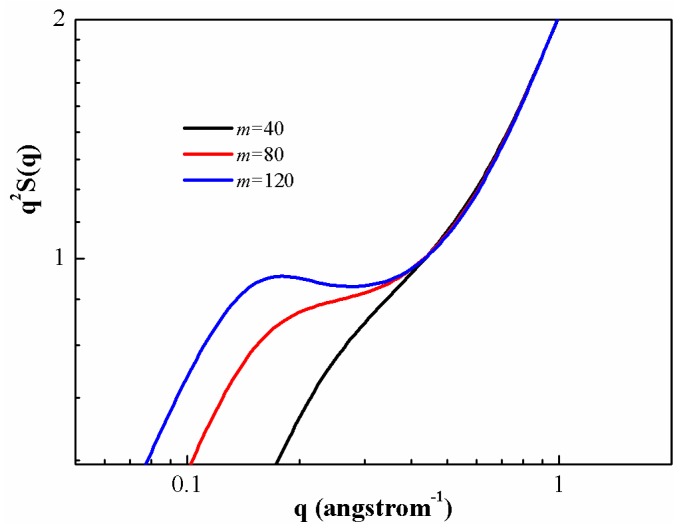
Structure factor of PS chain with three values of polymerization degree at 500 K.

(23) S(q)=ω(q)+ρh(q)=ω(q)+ρ[g(q)−1]

and the results for PS chains at different degrees of polymerization are presented in Figure 5. As can be seen, the general shape of the plots could be divided into three distinct regions. In the small wave vector regime (q≤0.2), S(q) is very sensitive to the degree of polymerization, both in magnitude and functional form. Increase of polymerization degree leads to enhanced S(q). The qualitative behavior of S(q) at small wave vectors is controlled by a competition between the intramolecular fluctuation and the intermolecular correlation. As the wave vector increases, there is a plateau-like regime (0.2≤q≤0.4) for S(q) (self-similar structure of the chains). This regime increases as the degree of polymerization increases. The plateau regime of high wave vectors is compound in nature, reflecting both the fixed bond length constraint and local intermolecular structure in the melt. In the high wave vector regime (q≥0.4), all the different PS melts have the same structure, *i.e.*, the same S(q).

**Figure 6:**
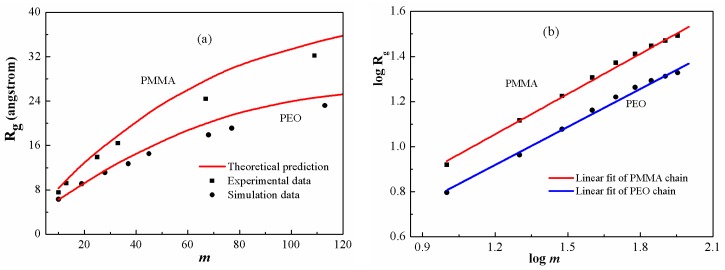
(**a**) Gyration radius as a function of chain length for PEO and PMMA melts at 300 K; (**b**) Determination of the scaling exponents for the two polymers. The squares and circles are calculated results and the solid line is the best linear regression.

Figure 6 shows the predicted gyration radii of PEO and PMMA chains in comparison respectively with the simulation [34] and experimental ones [49]. In Figure 6a, the theoretical predictions are generally in good agreement with the reported values. The results indicate that the current model is quantitatively reliable for describing the scaling properties in a broad range of polymeric systems. The scaling exponents for the two polymers with low polymerization are fitted in Figure 6b. The values are 0.59 and 0.56, which are smaller than the exponent for low-polymerized PS chains. The scaling exponents give an indication of stretching ratios. Among these three polymer melts, PS displays the maximum stretching ratio, whereas PEO has the minimum one. In this regard, the stretching ratio increases with enhancing chain stiffness.

**Figure 7:**
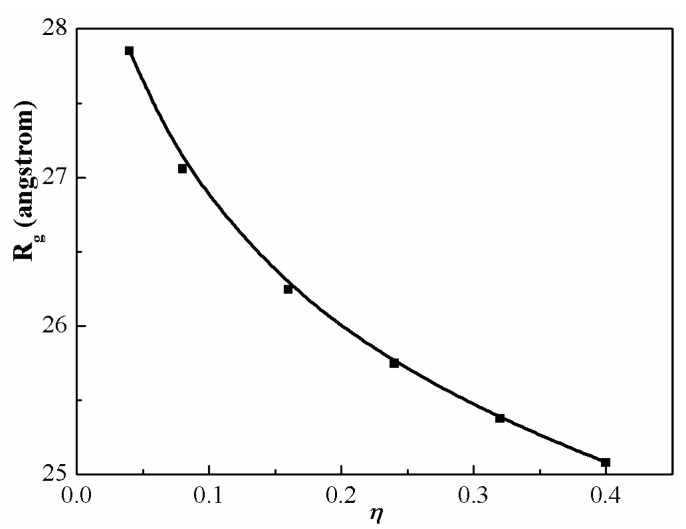
Gyration radius of PS chain as a function of packing fraction at 500 K. The chain length is fixed at m=100.

Figure 7 gives the gyration radii of PS chains with various packing fractions (η). Calculations are carried out for η in the range of 0.04 to 0.40. The highest packing mimics neat melt condition, while the lowest is more representative of a dilute solution. At low concentrations, the chains are extended but not rodlike. As the concentration increases, the size of PS chains decreases because of the greater screening of the intramolecular interactions. Another major result is that the change in averaged chain dimension is on the order of ~10% due to an incomplete cancellation of the long-range intra-chain excluded volume and condensed-phase-induced interactions. These modest nonideal effects are of the same qualitative size as discovered in diblock and triblock copolymer melts [50,51].

**Figure 8:**
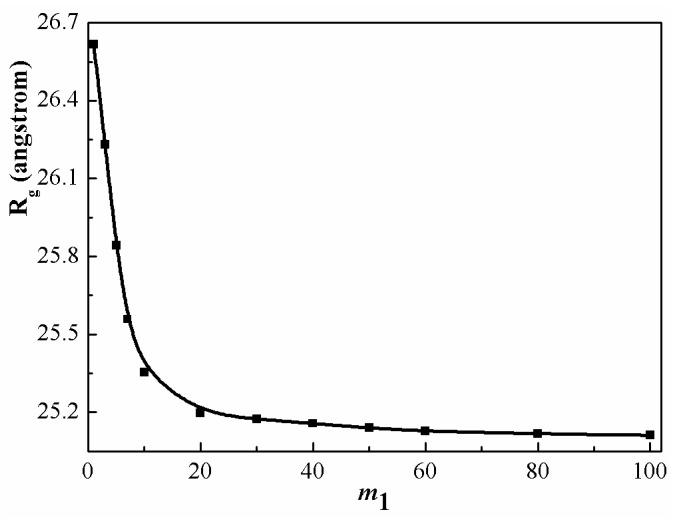
Gyration radius of long chain PS (m=100) as a function of chain length of short chain PS ($m_{1}=1-100$) at 500 K.

Finally, we consider the statistical properties of relatively long chains (polymerization degree m=100) immersed in a monodisperse melt of shorter, chemically identical chains (polymerization degree $m_{1}=1\sim 100$). Such systems can act as a fundamental test of the standard model for polymer conformation. Figure 8 describes the gyration radius of a long chain as a function of chain length of a short-chain molecule. If m is much larger than $m_{1}$, one sees that the short chain molecules acts as a good solvent and the long chains are swollen, owing to the excluded volume effect. As $m_{1}$ increases, the swelling extent of long chains decreases, since the excluded volume effects are screened by the surrounding polymer chains. The expansion of long chains becomes insignificant as the segment number of shorter chains is larger than a certain value ($m_{1}=40$). In the early theoretical investigations [9,15], it was predicted that polymer chains (with segment number m) can be expanded in its homologue (with segment number $m_{1}$) in the range of $m_{1}<m^{1/2}$. However, these investigations are based on an ideal chains model. Actual polymer chains have larger excluded volume. From the present theoretical model, we can find that the length range of $m_{1}$ has been enlarged for the stretching of PS chain when compared to the ideal chain blends.

## 4. Conclusions

We present a density functional approach to study the microscopic conformation of polymer chains. The form factors, structure factors, and gyration radii have been calculated based on the cited force field parameters of the coarse-grained model. Firstly, the effects of chain length to the scaling properties of PS, PMMA, and PEO melts have been compared to clarify the contribution of chain stiffness to the stretching ratios. The scaling exponents are 0.63, 0.59, and 0.56, respectively. The results reveal that polymer chains display non-universal behaviors due to their different stiffnesses. On the other hand, the effects of concentration on the stretching of PS chains have been calculated to evaluate the influence of medium. As the packing fraction of PS chain increases, the gyration radius of low polymerized PS declines. Finally, the swelling of PS chains blended with shorter PS chains has been analyzed, and an enlarged expansion regime has been observed, which is different from the ideal chain blends.

## Figures and Tables

**Table 1:** Bond and nonbond coarse-grained force field parameters.

Species	σ (Å)	ε(*k*)	θ(deg)	*L* (Å)
PS ^a^	5.08	62.5	140	2.46
PEO ^b^	4.30	405.8	130	3.30
PMMA ^c^	6.50	150.9	122	2.80

^a^ Reported in Reference [33]; ^b^ Reported in Reference [34]; ^c^ Reported in Reference [35].
